# The Added Value of the “Co” in Co-Culture Systems in Research on Osteoarthritis Pathology and Treatment Development

**DOI:** 10.3389/fbioe.2022.843056

**Published:** 2022-03-03

**Authors:** Katrin Agnes Muenzebrock, Valerie Kersten, Jacqueline Alblas, Joao Pedro Garcia, Laura B. Creemers

**Affiliations:** Orthopedics, University Medical Center Utrecht, Utrecht, Netherlands

**Keywords:** osteoarthirits, co-culture models, *ex vivo*, *in vitro*, tissue communication

## Abstract

Osteoarthritis (OA) is a highly prevalent disease and a major health burden. Its development and progression are influenced by factors such as age, obesity or joint overuse. As a whole organ disease OA affects not only cartilage, bone and synovium but also ligaments, fatty or nervous tissue surrounding the joint. These joint tissues interact with each other and understanding this interaction is important in developing novel treatments. To incorporate and study these interactions in OA research, several co-culture models have evolved. They combine two or more cell types or tissues and investigate the influence of amongst others inflammatory or degenerative stimuli seen in OA. This review focuses on co-cultures and the differential processes occurring in a given tissue or cell as a consequence of being combined with another joint cell type or tissue, and/or the extent to which a co-culture mimics the *in vivo* processes. Most co-culture models depart from synovial lining and cartilage culture, but also fat pad and bone have been included. Not all of the models appear to reflect the postulated *in vivo* OA pathophysiology, although some of the discrepancies may indicate current assumptions on this process are not entirely valid. Systematic analysis of the mutual influence the separate compartments in a given model exert on each other and validation against *in vivo* or *ex vivo* observation is still largely lacking and would increase their added value as *in vitro* OA models.

## 1 Introduction

Osteoarthritis (OA) is a degenerative and progressive joint disease affecting approximately 500 million people worldwide (Hunter et al., 2020). High age, obesity, a previous joint injury or chronic joint overuse are traits often connected with OA (Bijlsma et al., 2011) as is altered load distribution (Abramoff and Caldera, 2020). Within these phenotypes, inflammatory factors are variably involved in disease development and progression (Musumeci et al., 2015). The susceptibility to develop osteoarthritis can be increased by specific genetic alterations of proteins, for instance, involved in inflammatory processes or of components of the cartilage matrix (Sandell, 2012). Although a highly prevalent disease, current treatment options are mainly reducing the symptoms of OA: pain and loss of mobility, with pain management and physiotherapy as the main treatment strategies in primary treatment of OA (Hermann et al., 2018; Skou and Roos, 2019). As they fail to stop or reverse degenerative processes, prosthetic joint replacement is often the last resort in end-stage disease (Abramoff and Caldera, 2020).

OA is a whole organ disease affecting all tissues in and adjacent to the joint. The pathology of OA includes degradation of articular cartilage and ligaments, synovial inflammation (synovitis), malformation of subchondral bone and osteophyte formation. Adjoining muscles and nerves can also be affected by OA (Loeser et al., 2012; Schulze-Tanzil, 2019). The most investigated feature in OA treatment and research is articular cartilage degeneration. In healthy joints, cartilage forms a smooth surface that allows joint movement with very low friction (Johnston, 1997). In OA, the activity and phenotype of the resident chondrocytes are altered, with an increased activity of extracellular matrix degrading enzymes such as ADAMTS and collagenases causing structural and functional changes of the tissue (Goldring, 2000). Influx of immune cells into the synovium which forms the inner joint capsule is assumed to mediate cartilage degeneration by producing inflammatory mediators inducing production of matrix degrading enzymes in the cartilage and reducing synthetic activity (Pessler et al., 2008). The synovium also plays a role in OA pain development by promoting neurogenic inflammation mediated by neuropeptides such as substance P (Stanisz, 2001). The infrapatellar fat pad (IPFP), a tissue directly connected to the synovium, can be involved in OA progression as well. Cytokines and growth factors secreted from immune cells within the IPFP as well as adipokines produced by the adipose tissue increase the content of pro-inflammatory cytokines in the IPFP and in adjoining tissues (Klein-Wieringa et al., 2013). However, the infrapatellar fat pad might also play a beneficial role in joint health. An inverse relation between IPFP size and loss of joint space width in OA patients indicated a potential role of the IPFP as a shock absorber in the joint (Ioan-Facsinay and Kloppenburg, 2013; Han et al., 2014). Finally, a clear interaction between bone and cartilage is present. An increase of TGF-β in subchondral bone, for instance, was found to decrease proteoglycan content in the adjacent cartilage and mediate the OA development in an ACLT mouse model (Zhen et al., 2013). Microcracks and fissures in the subchondral bone are thought to facilitate exchange of molecules between bone and cartilage in human osteochondral explants from OA patients, e.g. by increased hydraulic conductance (Hwang et al., 2008; Yuan et al., 2014). Increased vascularization of the subchondral bone due to OA is also thought to increase the exchange (Yuan et al., 2014). Taken together, the influence as well as the interconnection of different joint compartments on OA pathogenesis has been clearly demonstrated.

Although for final proof of the relevance of these interactions commonly *in vivo* studies are carried out, recently the interest in the use of *in vitro* models to replace *in vivo* studies in joint research has intensified, especially against the backdrop of the societal demand to reduce animal use (Ormandy and Schuppli, 2014). Several types of *in vitro* models are available [elegantly reviewed by Piluso et al. (2019)], of which co-cultures of different cells or tissues are most suited to address the role of interaction between joint tissues in OA pathophysiology. Studying the connection between the different cell types and joint structures could further improve the understanding of OA development and progression. Consequently, the development of therapies for patients suffering from joint diseases would be enhanced. This review will therefore discuss co-culture systems with a focus on their potency to address tissue interaction and their use in drug development in OA. Many co-culture models are used based on the assumption that the mere combination of tissues or cells results in an interaction between the cells in these tissues, and that this interaction reflects OA processes *in vivo*. In our review we therefore limited our overview of co-culture systems to those in which a clear interaction was demonstrated through differential behaviour of tissues or cells caused by the presence of the other tissue(s)/cells, and/or if a culture system was shown to reflect *in vivo* pathophysiological phenomena. Also co-cultures combining cells or tissues as part of regenerative approaches (e.g. MSCs and chondrocytes) were excluded.

## 2 Cell-Based Two-Dimensional Co-culture Models

**FIGURE 1:**
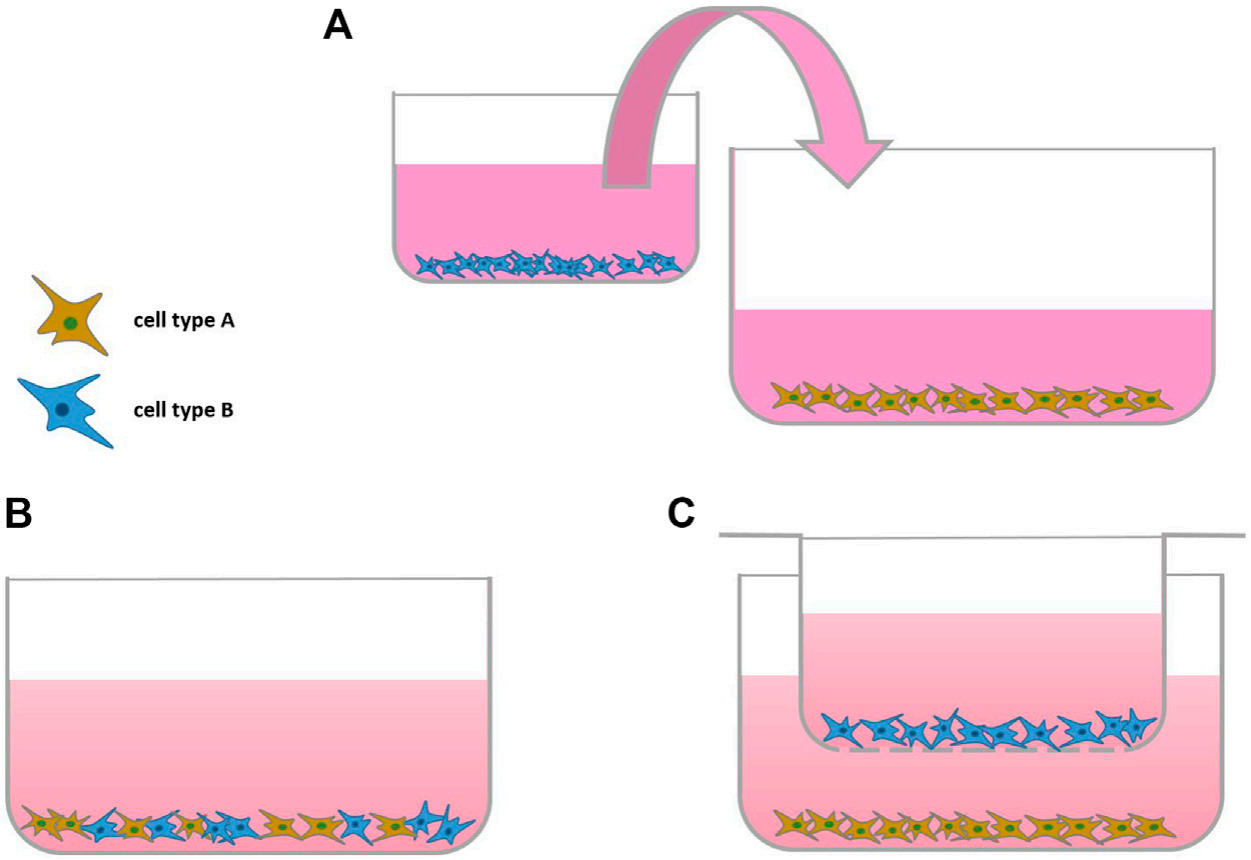
Scheme of Monolayer Co-cultures: **(A)** indirect co-culture, **(B,C)** direct co-culture without and **(C)** with using a cell-culture insert (transwell).

The interplay of different cell types can be investigated using co-culture. Indirect co-culture models, so culturing one cell type in conditioned medium of another cell type of interest can provide valuable insights. To add more complexity to a monolayer culture, a second cell type is added to form a direct co-culture system. Cells can either be mixed directly and seeded into a monolayer culture or separated using cell culture inserts (Figure 1). A benefit of co-culture models is that they are able to demonstrate cell-cell interaction of different cell types involved in OA onset or progression (see Table 1). Ideally, cell-based co-culture departs from primary cells. Although cell lines are easier to access and handle, they sometimes lack traits of primary cells, such as the production of inflammatory factors upon stimulation (Santoro et al., 2015).

**TABLE 1:** Summary of the effects within different co-culture models in monolayer.

Model	Cell	Cell origin	Effect of co-culture	Additional stimulus	Effect on monoculture	Effect on co-culture	References
ACL fibroblasts & synovial fibroblasts	ligament fibroblasts	human	lysyl oxidase ↓	TNFα	MMPs ↑	MMPs ↑↑	Wang et al. (2019)
				TNFα + mech. stress	MMPs ↑	lysyl oxidase ↓↓	
chondrocytes & synovial fibroblasts	chondrocytes	human		IL-1β		H3Ser10 phosphorylation, NFκB activity ↑	Pagani et al. (2019)
chondrocytes & OA osteoblasts	chondrocytes	human	MMPs ↑, ADAMTS-4,-5 ↑ by conditioned medium				Prasadam et al. (2012)
chondrocytes & mononuclear (MN) cells	chondrocytes	human	MMPs ↑, ADAMTS-4,-5 ↑				Platzer et al. (2020)
	MN cells	Human	not described				

Conditioned medium of osteoarthritic osteoblasts could increase activity and expression of matrix degrading enzymes such as ADAMTS-4, ADAMTS-5 or various matrix metalloproteases (MMPs) in non-arthritic chondrocytes compared to chondrocytes in conditioned medium from non-arthritic osteoblasts or medium only (Prasadam et al., 2012). The influence of peripheral blood mononuclear cells (PBMC) in osteoarthritic joints was studied by co-culture of human chondrocytes with CD4^+^CD127^dim/-^ enriched PBMCs. A significant increase of MMP-1 and ADAMTS-5 was found upon co-culture, compared to chondrocytes cultured alone (Platzer et al., 2020).

As rupture of anterior cruciate ligaments (ACL) can result in OA development, the impact of synoviocytes on ligament fibroblasts upon stimulation with TNFα and/or mechanical stress was investigated (Wang et al., 2019). Synoviocytes and ACL fibroblasts obtained from patients undergoing knee replacement after an accident were co-cultured using culture inserts. While TNF-α stimulation slightly increased MMP-1, -2, and -3 and decreased lysyl oxidase (LOX) expression, a marker for ligament healing, these effects were amplified by addition of synoviocytes to the injured ACL fibroblasts. Hence, in this model, the behavior of synoviocytes seems to be mainly inflammatory (Wang et al., 2019). To what extent the synoviocytes of acutely injured joints can be considered as diseased and therefore the phenomena observed match those occurring *in vivo* in OA, cannot be verified with certainty, however.

## 3 Cell-Based Three-Dimensional Co-culture Models

**FIGURE 2:**
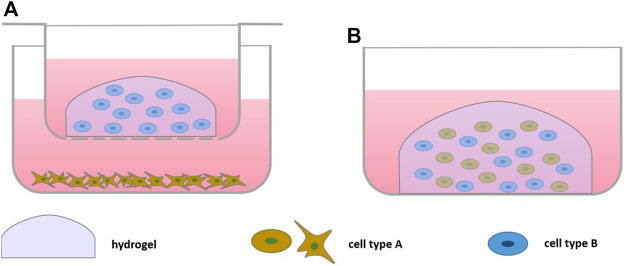
Scheme of Bioengineered Cell-based Co-culture: **(A)** direct co-culture embedding one cell type in hydrogel in a culture insert on top of a monolayer culture, **(B)** direct co-culture embedding both cell types in a hydrogel.

2D culture fails to recapitulate the three-dimensional organization of cartilage and other tissues of the joint. Therefore, usually 3D cultures of chondrocytes or cells encapsulated into hydrogels are utilized. Both natural and synthetic hydrogels are used for the bioengineering of tissues (Table 2) (Figure 2). They are thought to mimic the physiological environment and support viability, proliferation and secretory abilities (Lee and Mooney, 2001; Vinatier and Guicheux, 2016) especially from chondrocytes. Care must be taken in their selection, however, as biomaterials affect cell phenotype (Tsuchida et al., 2013), which may be in part due to hydrogel biomechanical properties and the possibility for material-cell interaction (Engler et al., 2006; Krouwels et al., 2018).

**TABLE 2:** Summary of the effects within different cell-based bioengineered co-culture models.

Model	Hydrogel	Cell origin	Cell	Effect of co-culture	Additional stimulus	Effect on monoculture	Effect on co-culture	References
MSOD & HUVEC	GelMA- based	human	MSOD	osteogenic differentiation	a) IL-1β, TNF-α, IL-6	ALP↑	ALP↑↑, mineralization ↓	Pirosa et al. (2021)
					b) cond. chondrocyte medium		mineralization ↓	
			HUVEC		a) IL-1β, TNF-α, IL-6	VEGF↑ endoth. network formation	VEGF ↑↑, endoth. network maintenance ↑	
					b) conditioned chondrocyte medium		no network formation	
OA chondrocytes activated macrophages	PEGDA	human	chondrocytes	MMPs ↑, IL-1β, TNF-α, IL-6, IL-8 and IFN-γ ↑				Samavedi et al. (2017)
		murine	macrophages	IL-1β and Arginase-1 ↑				
chondrocytes & activated macrophages	gelatin	porcine	chondrocytes	MMPs ↑, coll II and aggrecan exp.↑; proliferation ↑, coll II GAG content ↓				Peck et al. (2014)
		murine	macrophages	not described				
chondrocytes & osteoblasts	alginate	human	chondrocytes		a) human sclerotic osteoblasts		MMP-3, ADAMTS-4,-5 ↑ SOX-9 and coll II ↑, aggrecan ↓	Sanchez et al. (2005a), Sanchez et al. (2005b), Lin et al. (2010), Sanchez et al. (2015)
					b) IL-1β + IL-6 or OSM		MMPs ↑, aggrecan ↓	
		porcine	chondrocytes	hypertrophy (coll II, aggrecan↓; coll X, bone sialoprotein ↑)	mech. stress		MMPs, ADAMTS-4, -5 ↑, hypertrophy ↑↑	
		a) or b)	osteoblast	not described				
chondrogenic and osteogenic diff. hBMSC	GelMA-based bio-reactor	human	chondrogenic cells		IL-1β	MMPs↑	MMPs↑↑	Lozito et al. (2013), Lin et al. (2014)
chondrocytes and synovial fibroblasts	alginate	murine	chondrocytes		IL-1β		proteoglycan ↓, NO and PGE_2_ ↑	Gouze et al. (2004)
			syn. fibroblast	not described				
joint on a chip	fibrin	human	HUVEC		a) TNFα + chemokines		monocyte extravasation ↑	Mondadori et al. (2021)
							+ chemokines ↑↑	
					b) OA synovium		monocyte extravasation ↑↑	
			OA chondrocytes	not described				
			syn. fibroblasts	not described				

### 3.1 Chondrocyte and Monocytes/Macrophages

Co-culture with macrophages reflects the interaction of the type B cells in the synovial lining with cartilage. Upon addition of a LPS-activated murine macrophage cell line to human OA chondrocytes cultured in poly-(ethylene glycol)-diacrylate (PEGDA) using culture inserts, a significant increase of MMP-1, MMP-3, MMP-9, MMP-13, IL-1β, TNF-α, IL-6, IL-8, and IFN-γ compared to OA chondrocytes in monoculture was observed (Samavedi et al., 2017). Conversely, in macrophages, IL-1β and Arginase-1, a macrophage inflammatory marker, were significantly increased by the presence of the chondrocytes. This suggests that changes induced by OA in either cell type intensified response in the neighboring cell type, highlighting the use of such cultures to study the crosstalk of macrophages and chondrocytes in OA (Samavedi et al., 2017). The catabolic marker panel found was similar to that observed *in vitro* OA models, indicating the validity of the model (Blasioli et al., 2014; Samavedi et al., 2017). Whether the effect of OA chondrocytes on activated macrophages was specific for the OA state is not clear, as healthy chondrocytes were not included in the study. Addition of LPS- activated and non-activated murine macrophages to gelatin-embedded porcine chondrocytes increased chondrocyte expression of MMP-1, MMP-3 after 1 week of co-culture (Peck et al., 2014). Activated macrophages also increased cell proliferation as well as collagen II and aggrecan expression, which was claimed to mimic anabolism in early OA. However, at the protein level total GAG and collagen II content were significantly reduced upon co-culture with both unstimulated and LPS-stimulated macrophages. Celecoxib treatment of the co-culture with LPS-activated macrophages was able to significantly reduce MMP-1 and MMP-3 expression after 3 days. PGE_2_ levels were significantly reduced after 7 days of treatment indicating that the model might be applicable for drug testing (Peck et al., 2014). The inhibition of chondrocyte ECM synthesis by non-stimulated macrophages however, and the combination of murine with porcine cells should be viewed critically.

### 3.2 Chondrocytes and Bone Cells

Focusing on the interaction between cartilage and bone, several models incorporating bone cells have been described, many of these based on alginate-encapsulated chondrocytes in culture inserts and monolayers of osteoblasts (Lin et al., 2010; Sanchez et al., 2005a; Sanchez et al., 2005b; Sanchez et al., 2015) (Figure 2). Using healthy tissue as source, porcine osteoblasts induced chondrocyte hypertrophy as shown by decreased collagen II and aggrecan expression and increased expression of collagen X and bone sialoprotein (Lin et al., 2010), factors involved in OA pathology (Pesesse et al., 2014). If osteoblasts were subjected to cyclic tensile stress, an even more distinct shift towards hypertrophy and additional increase of MMP-1, MMP-3 and MMP-13 expression in chondrocytes was observed, supporting the view that mechanical stress of bone could induce degenerative changes in cartilage. *In vivo,* mechanical stress of bone lead to increased TGFβ signaling, which in turn could promote OA (Zhen and Cao, 2014), matching the findings in this *in vitro* model. TGFβ expression was also increased in the stressed osteoblasts in the model (Lin et al., 2010). Still, the observation that healthy osteoblasts can also induce OA-like changes may indicate less validity of this model.

Human osteoblasts also decreased SOX-9 and collagen II gene expression of human chondrocytes, which was more pronounced by addition of sclerotic compared to non-sclerotic osteoblasts (Sanchez et al., 2005a). Fibroblasts did not induce these changes in chondrocytes, indicating that this influence might be specific to the communication of chondrocytes and osteoblasts (Sanchez et al., 2005a). Pretreating non-sclerotic osteoblasts with an inflammatory stimulus (IL-1β + IL-6 + soluble IL-6 receptor or oncostatin M (OSM)) at levels found in OA synovial fluid upregulated MMP expression and downregulated aggrecan expression to similar extent as the sclerotic osteoblasts (Sanchez et al., 2005b). Although also here healthy cells negatively affected chondrocyte behavior, the more pronounced effects of diseased osteoblasts, in line with *in vivo* observations, suggest its applicability as culture model.

**FIGURE 3:**
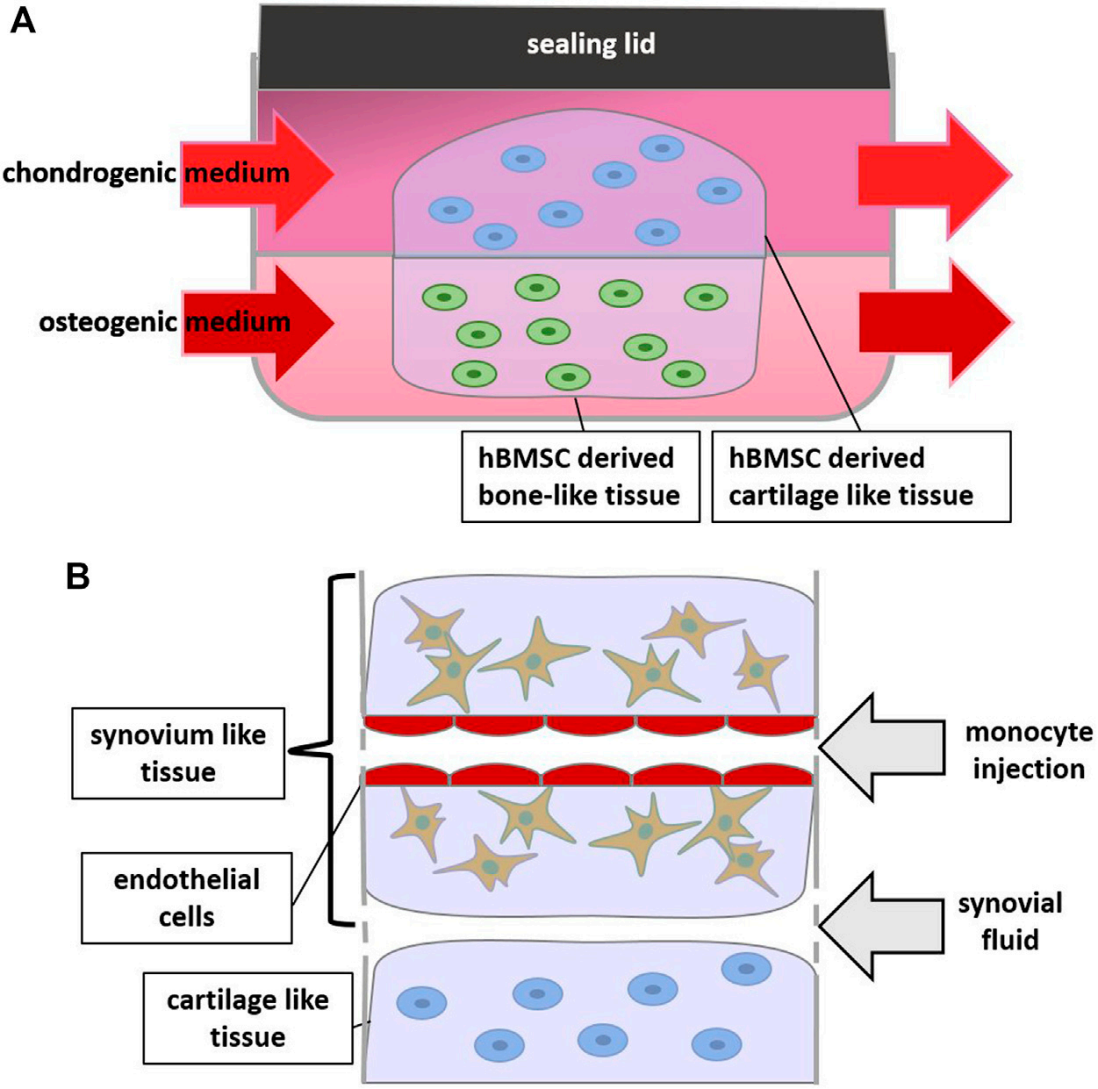
Scheme of Bioreactors and Joint-on-a-Chip: **(A)** 2 hydrogels containing chondrogenic and osteogenic differentiated cells respectively are supplied by different media, **(B)** zoom in on a joint-on-a-chip containing synovium, cartilage and endothelium-like structures which allows for monocyte extravasation experiments.

Also in a microsystem bioreactor using microfluidics (Lozito et al., 2013) (Figure 3A), the influence of IL-1β stimulation on osseous and cartilage-like tissue components was studied. This model demonstrated that stimulating hBMSC-generated bone-like tissue with IL-1β resulted in a greater inflammatory response (e.g., increased expression of MMPs) in the adjoining cartilage-like component than by stimulating the cartilage component directly, suggesting communication between both joint compartments (Lin et al., 2014).

### 3.3 Chondrocytes and Synovium Cells

A bioengineered co-culture model of chondrocytes and synovial fibroblasts was used to investigate whether overexpression of glutamine fructose-6-phosphate amidotransferase (GFAT), an enzyme involved in glucosamine production, can influence matrix production. Rat synovial fibroblasts were adenovirally transduced with GFAT cDNA, co-cultured with alginate-encapsulated chondrocytes and stimulated with IL-1β to investigate the influence of GFAT on the response to IL-1β (Gouze et al., 2004). While in the non-transduced co-culture control, a decrease of proteoglycan production in chondrocytes and a simultaneous increase of nitric oxide and PGE_2_ in the medium were observed after IL-1β stimulation, GFAT overexpression in synoviocytes prevented these changes (Gouze et al., 2004). Thereby the study demonstrated that a co-culture model can be utilized to study how gene therapy in one tissue can affect an adjoining tissue in OA.

Complex systems can nowadays easily be reconstructed in organ-on-a-chip approaches. Monocyte extravasation in response to chemokines or the synovial fluid in OA was studied in a joint-on-a-chip-model including OA patient-derived synovial fluid, fibrin hydrogel-embedded OA chondrocytes and OA synovial fibroblasts, as well as perfusable endothelialized channels for monocyte injection (Mondadori et al., 2021) (Figure 3B). To mimic shear stress present in the channel, laminar flow of the medium was applied. This induced a shift towards more physiological expression levels of endothelial markers VCAM and ICAM, demonstrating the ability to mimic the *in vivo* situation to a certain extent. Additional TNFα treatment of the endothelial cells synergistically increased ICAM expression. Addition of a chemokine mix (CCL 2, CCL 3, CCL 4, and CCL 5) to the synovial fluid-mimicking compartment further stimulated monocyte extravasation. OA synovial fluid was similarly able to increase monocyte extravasation significantly, suggesting that synovial fluid might be relevant for monocyte extravasation *in vivo* (Mondadori et al., 2021). This model was used to test a CCR2 chemokine receptor antagonist and an antagonist for chemokine receptors CCR2 and CCR5. The antagonists reduced monocyte extravasation, showing that the model could be utilized for drug testing. The effects on the cartilage compartment was unfortunately not further investigated, nor was the added value of any of the other compartments investigated.

### 3.4 Bone Cells and Endothelial Cells

In order to create an OA model for subchondral bone and its vasculature, a photo cross-linked gelatin methacrylate (GelMA)-based co-culture of a mix of immortalized mesenchymal stromal cell line (MSOD) and HUVEC cells was utilized. MSOD cells underwent osteogenic differentiation by the presence of the HUVEC cells (Pirosa et al., 2021) MSOD mono and co-culture showed signs of mineralization. Cytokine stimulation (IL-1β, TNF-α and IL-6, all at concentrations found in synovial fluid of OA patients) increased endothelial network formation, similar to what is found in OA, in both HUVEC and MSOD-HUVEC culture. However, addition of the MSOD was required to maintain the network. Stimulation also induced demineralization and increase of collagen synthesis similar to changes in OA bone (Pirosa et al., 2021). Moreover, the cytokine-induced increased expression of alkaline phosphatase (ALP) and vascular endothelial growth factor (VEGF), markers for osteogenesis and angiogenesis, respectively, was more pronounced in co-culture. The co-culture was also stimulated with conditioned medium of a cartilage-on-a-chip model which consisted of chondrocytes that were embedded in a PEG-based hydrogel loaded onto a compressible PDMS device (Pirosa et al., 2021). Cells in the chip were supra-physiologically compressed to induce an osteoarthritic phenotype (Occhetta et al., 2019). Stimulation of the MSOD-HUVEC co-culture with the conditioned medium induced demineralization but prevented endothelial network formation, suggesting the pathways triggered were distinctly different from those induced by the abovementioned cytokine panel (Pirosa et al., 2021). Taken together co-culture could clearly demonstrate crosstalk between both cell types and also indicates that bone-like cells influence the phenotype of endothelial cells and vice versa. However, to what extent the MSODs really differentiated into bone cells was not clear.

## 4 Co-Cultured Tissue Explants

**FIGURE 4:**
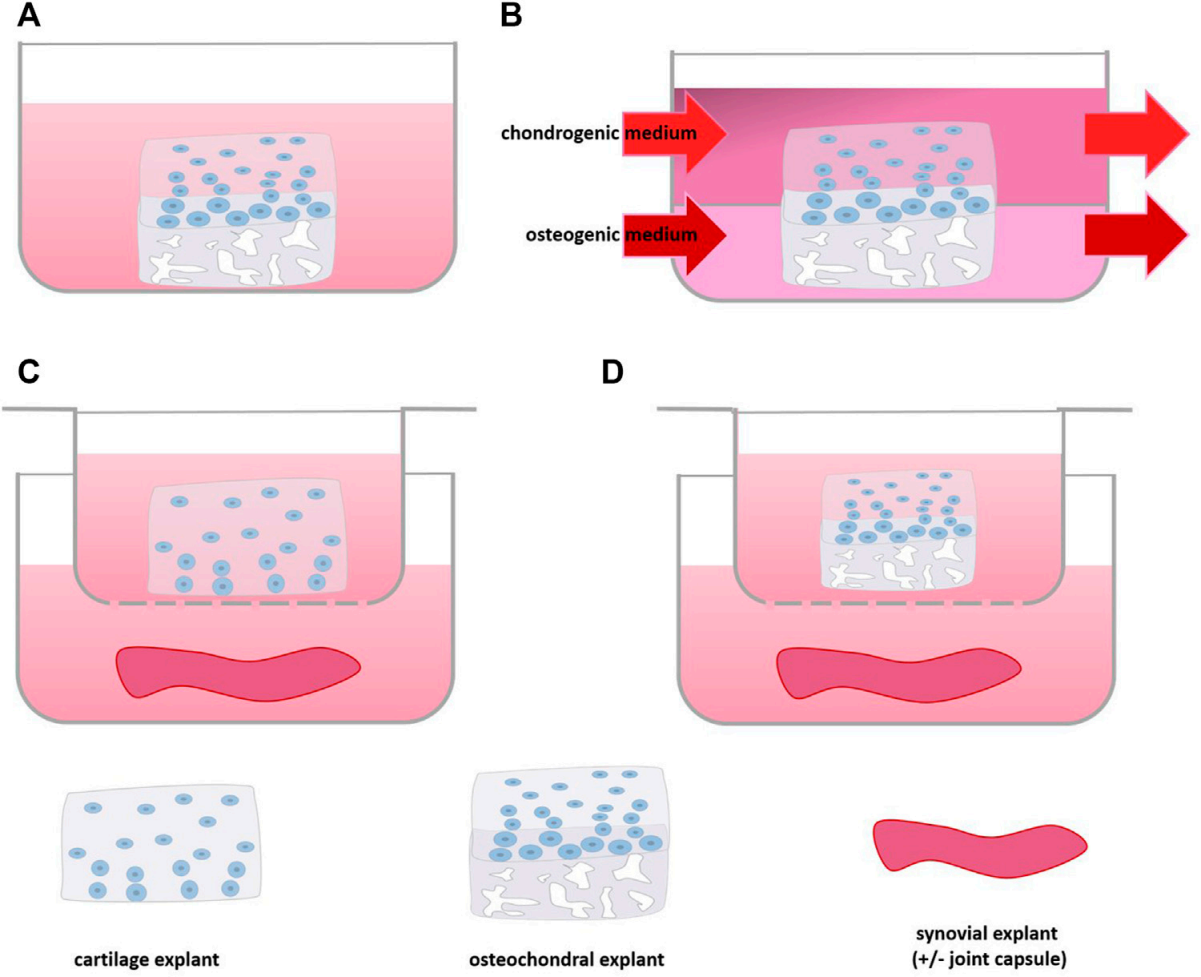
Scheme of Tissue Explant Co-culture: **(A)** culture of osteochondral tissue, **(B)** with separate medium supply for cartilage and bone part, **(C)** co-culture of synovium and cartilage or **(D)** osteochondral tissue using a culture insert.

**FIGURE 5:**
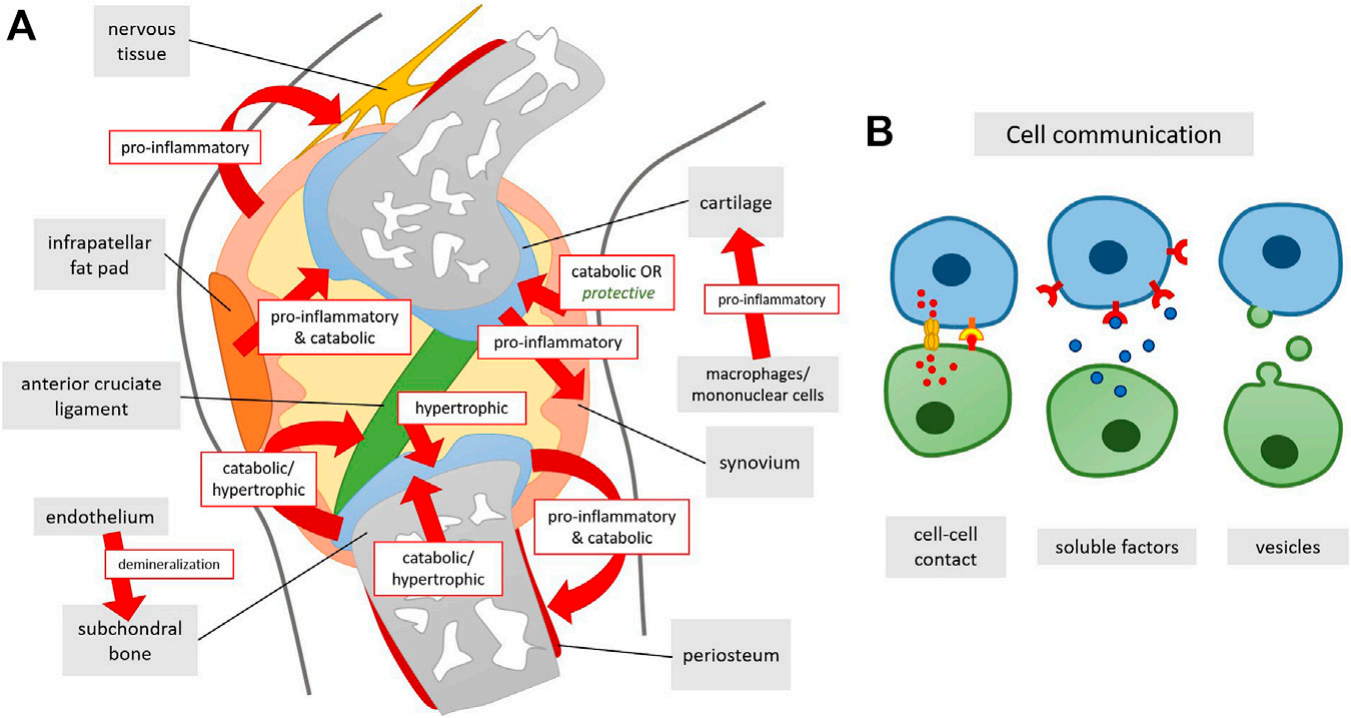
**(A)** Summary of co-culturing effects on tissue level in the knee joint and **(B)** underlying ways of cellular communication. Arrows indicate the effect of one of the tissues/cell types onto the tissue it is directed at.

Although cell-based co-culture is a versatile strategy, one of the clear disadvantage is the change in phenotype occurring as a consequence of isolation and expansion of primary cells (Zimmermann et al., 2000; Schnabel et al., 2002; Tsuchida et al., 2014; Rosenzweig et al., 2017). In order to improve translation from model to clinic by studying cell behavior in their natural habitat, tissue explant models have been used in which the physiological or pathological microenvironment of the cells inside is maintained (Geurts et al., 2018). In OA research, these are often derived from cow, but also horse, dog or sheep (Greenberg et al., 2006; Lee et al., 2013; Byron and Trahan, 2017; Swärd et al., 2017; Haltmayer et al., 2019; Mehta et al., 2019), and also human explants gained importance in OA research (Table 3) (Hardy et al., 2002; Schwab et al., 2017; Geurts et al., 2018; Topoluk et al., 2018; Favero et al., 2019; Houtman et al., 2021a; Houtman et al., 2021b). Cartilage tissue co-culture models mainly comprise explants of cartilage combined with primary cells or other joint components, such as the attached subchondral bone (Byron and Trahan, 2017; Schwab et al., 2017) synovium (Hardy et al., 2002; O'Brien et al., 2019; Osterman et al., 2015; Araújo et al., 2020) or joint capsule (Swärd et al., 2017), the infrapatellar fat pad (Nishimuta et al., 2017) or nervous tissue (Li et al., 2011) (Figures 4, 5A).

**TABLE 3:** Summary of effects within different tissue explant-based co-culture models.

Model	Cell/tissue origin	Cell/tissue	Effect of co-culture	Additional stimulus	Effect on monoculture	Effect on co-culture	References
cartilage & synovial fibroblasts	equine	cartilage		a) mechanic. stress	cell clusters & focal cell loss↓	coll II ↑ aggrecan ↑	Gregg et al. (2006), Lee et al. (2013)
				b) IL-1β		GAG loss ↓	
	equine	synovial fibroblasts		mechanical stress		ADAMTS-4,-5 ↓, MMP-1 ↑, MMP-3 ↓	
chondrocytes & synovium	rat	chondrocytes		injury on synovium		early OA: aggrecan ↑, late OA: MCP-1 ↑	Lai-Zhao et al. (2021)
	rat	synovium	not described				
damaged ACL & chondrocytes	human	chondrocytes		a) acute damaged ACL		coll II ↓ and ADAMTS-4 ↑ periostin ↑	Chinzei et al. (2018)
				b)chronic damaged ACL		col II ↑ MMP-13 and ADAMTS-4 ↑ periostin ↑ lL-1 ↓	
periosteum & chondrocyte pellets	human	periosteum	COL1A1 ↑, TGF-β↑				Grässel et al. (2010), Rickert et al. (2010), Steinhagen et al. (2012)
			IL-6, MMP-2, -7, -13 ↑				
	bovine	periosteum	coll II deposition↓				
			GAG synthesis & release ↓				
	human	chondrocytes	collagen I deposition				
cartilage & synovium	canine	cartilage	maintained proteoglycan content	IL-1β	MMP-13 ↑	proteoglycans↓ gene expression e.g. coll II & MMPs closer to OA patient material	Hardy et al. (2002), Cook et al. (2007), Beekhuizen et al. (2011)
	canine	synovium				COX-2, PGE_2_ ↑	
OA cartilage & OA synovium	human	cartilage	MMP-13 ↑, cell viability ↓, GAG production ↓, (GAG release ↑)				
	human	synovium	not described				
cartilage & joint capsule	bovine	cartilage	MMP-13, ADAMTS-4 ↑	mechanical injury		aggrecan digestion↑ MMP-3, ADAMTS-4, -5↑	Lee et al. (2009), Swärd et al. (2017)
	bovine	joint capsule	not described				
osteochondral & synovium	equine	osteochondral explant	collagen II ↑	a) IL-1β	TNF-α, MMP-13 ↑↑	TNF-α, MMP-13 ↑	Byron and Trahan. (2017), Haltmayer et al. (2019)
				b) mech. injury, IL-1β and TNF-α	MMP-1 ↑	MMP-1 ↑↑	
	equine	synovium		b) mech. injury, IL-1β and TNF-α		macrophage shift → M1	
meniscus & OA synovium	human	meniscus	IL-6, IL-8 ↑, MMP-3,-10 ↑, GAG release ↑				Favero et al. (2019)
	human	synovium	not described				
osteochondral	human	osteochondral explant		a-c) respectively		MMP-13 & HIF-2α ↑	Geurts et al. (2018), Houtman et al. (2021a), Houtman et al. (2021b)
				a) IL-1 β		COL1A1 ↑	
				b) mech. injury		senescence markers ↑	
				c) triiodothyronine		COL2A1 ↓	
				d) LPS		IL-6, MCP-1 ↑	
dorsal root ganglia & OA synovium	rat	dorsal root ganglia	neurokinins, neuropeptide Y ↑				Li et al. (2011)
	human	OA synovium	not described				
cartilage & fat	bovine	cartilage	GAG release ↑				Nishimuta et al. (2017); Zhou et al. (2020)
	human	cartilage		OA IPFP cond. medium		collagen & proteoglycan loss	
						MMP-3, COX-2↑	
		chondrocytes		OA IPFP cond. medium		p38MAPK and ERK1/2↑, MMPS, ADAMTS-4 ↑, IL-1β, IL6 and COX-2 ↑	
	either	fat	not described				

### 4.1 Co-culture of Explants and Primary Cells

Upon exposure to mechanically injured equine cartilage, primary equine healthy synovial fibroblasts reduced their ADAMTS-4 and 5 expression while increasing expression of MMP-1 as compared to co-culture with normal cartilage (Lee et al., 2013). Vice versa the fibroblasts induced higher collagen II expression in the injured cartilage and also histologically the progression towards an OA phenotype in injured cartilage seemed inhibited, evidenced by decreased cell clusters and focal cell loss (Lee et al., 2013). Also in IL-1β-stimulated equine cartilage, synoviocytes reduced GAG loss and diminished downregulation of aggrecan, although MMP-3 in synoviocytes was upregulated (Gregg et al., 2006). Altogether, synovial cells seem to exert a protective effect on cartilage. To what extent this is found *in vivo* is not clear.

In an elegant study where healthy rat chondrocytes were co-cultured with synovium harvested at different time points after OA induction, addition of synovium initially showed higher aggrecan production of the chondrocytes, suggesting injury induced a transient anabolic effect. With increased OA stage of the isolated synovium, however, this changed into an increase of monocyte chemoattractant protein 1 (MCP-1), a chemokine initiating inflammation (Xu et al., 2015; Lai-Zhao et al., 2021). The effects did not seem to be related to the OA induction surgery, but rather the injury of the joint, as similar results were observed using synovium from sham surgery controls (Lai-Zhao et al., 2021). A similar approach of determining the effects on joint tissue interaction of early pathological processes involved in OA development was taken by adding human ACL explants from joints with acute and chronic ACL damage to chondrocytes culture. Both types of explants increased human chondrocyte periostin and ADAMTS-4 expression, chronic remnants reduced Il-1, and increased collagen II and MMP-13 expression, whereas explants from acutely injured ACL decreased collagen II expression. It must be noted that only effects at the mRNA level were studied, and hence may not entirely reflect the metabolic state of the chondrocytes (Chinzei et al., 2018). Although applied as part of the search for cartilage regenerative strategies, a co-culture model of pellets of human chondrocytes from preserved areas in OA cartilage and periosteum explants separated by a culture insert can also be used to mimic the paracrine interaction of these tissues in the joint. Addition of chondrocytes significantly increased TGF-β and COL1A1 gene expression and induced expression of IL-6, MMP-2, -7, and -13 expression in periosteum. The periosteum induced collagen I deposition in pellets (Grässel et al., 2010; Rickert et al., 2010). In contrast, the addition of healthy bovine periosteum increased proliferation and decreased collagen II matrix deposition as well as aggrecan synthesis and release in chondrocyte monolayer, suggesting an overall inhibitory effect of periosteum (Steinhagen et al., 2012).

### 4.2 Osteochondral Explants

Osteochondral explants consist of cartilage attached to the underlying subchondral bone (Figure 4A). Although in this culture system it is not possible to point out the added value of one or the other tissue in the model, it is a frequently used model that has shown to reflect many aspects of *in vivo* disease (Houtman et al., 2021a; Houtman et al., 2021b). A transcriptome wide analysis of mechanically stressed human osteochondral explants demonstrated an increase of MMP-13 gene expression (Dunn et al., 2016; Houtman et al., 2021a). Moreover, increased expression of senescence markers such as FOXO and MYC1 were found after mechanical stimulation, which have also been found to be dysregulated in OA chondrocytes *in vivo* (Matsuzaki et al., 2018; Wu et al., 2018) indicating that the model harbors features of OA. In a study on finding a suitable culture system for OA, conventional stimulation of osteochondral explants by IL-1β was compared to triiodothyronine (T3) stimulation and mechanical stress (Houtman et al., 2021b). On the one hand all three stimuli induced an increase of HIF-2A and MMP-13 and no change in ADAMTS-5 gene expression. On the other hand only IL-1β reduced COL2A1 expression, while T3 stimulation increased COL1A1 expression and hypertrophy, while the mechanical loading only altered mechanical properties of the explant. Thus, depending on the stimulus, but most likely also its dose, different changes in the osteochondral explant can be induced that mimic the different disease aspects of OA (Houtman et al., 2021b).

The effect of Toll-like receptor 4 (TLR-4) agonist lipopolysaccharide (LPS) was tested on human osteochondral explants to see whether it could be used to create an OA model for drug testing. LPS activates TLR-4 in a similar fashion as damage-associated molecular patterns (DAMP) which are thought to play a key role in the inflammatory processes in OA (Geurts et al., 2018; Lambert et al., 2021). In the model, LPS induction increased secretion of the inflammatory markers IL-6 and MCP-1 (Geurts et al., 2018). A similar increase of IL-6 and MCP-1 was also found in LPS-stimulated bone explants, suggesting that the effects seen in the osteochondral explants involved the osseous compartment of the osteochondral explant. Treatment of the LPS stimulated osteochondral explant with SB-505124, a TGF-β receptor type I inhibitor that was shown to attenuate cartilage degradation *in vivo* (Zhang et al., 2018), could reduce IL-6 secretion but increased MCP-1 secretion into the medium (Geurts et al., 2018). Unfortunately, the effects on cartilage integrity were not studied to verify whether these coincided with previous *in vivo* findings. Of note, in the described models the osteochondral explants were cultured in the same culture medium. However, bone cells require other culture conditions than chondrocytes. Using either chondrogenic or osteogenic medium for osteochondral explants precludes optimal culture of both tissue compartments (Schwab et al., 2017). Moreover, the bone tissue may secrete cytokines and other substances into the medium at non-physiological levels (Sanchez et al., 2005a; Iwai et al., 2011). A co-culture platform for osteochondral explants consisting of two separated media compartments was developed to overcome this drawback (Figure 4B). A porcine model proved that the platform enabled supply of tissue specific medium and factors. Here, osteochondral explants could be cultured for at least 8 weeks maintaining matrix content and structural and mechanical properties without decreasing viability (Schwab et al., 2017). Combining this culture set up with the aforementioned stimuli could lead to a better OA model where both bone and cartilage could be investigated in a setup closer to the *in vivo* situation.

### 4.3 Cartilaginous & Synovium Tissue

Also the crosstalk between cartilaginous tissue and synovium, where the release of inflammatory cytokines by the inflamed synovium has been considered a major driver of the production of matrix degrading enzymes by the cartilage, was addressed in co-culture systems ( Cook et al., 2007; Lee et al., 2009; Beekhuizen et al., 2011; Topoluk et al., 2018; Favero et al., 2019; Lai-Zhao et al., 2021). Adding healthy synovial tissue to canine cartilage explants (Figure 4C) maintained Toluidine blue staining indicative of proteoglycan content to *in vivo* levels, whereas in cartilage monoculture staining was more faint, suggesting proteoglycan loss (Cook et al., 2007). IL-1β stimulation induced a significant increase of both nitric oxide as well as prostaglandin E_2_ (PGE_2_) in culture medium of co-cultures, but not in cartilage monoculture. Moreover, MMP-13 secretion was increased to a higher extent after IL-1β stimulation in co-culture compared to cartilage monoculture, in line with the postulated role of the inflamed synovial lining *in vivo*. Gene expression of collagens, aggrecan and catabolic enzymes (MMPs, ADAMTS-4 and 5) of canine OA cartilage in co-culture with OA synovial tissue was more in line with tissue directly derived from OA patients than of cartilage in monoculture (Cook et al., 2007). Also in cultures of human OA cartilage and synovial tissue, a series of cytokines were produced that matched the expression profile seen in synovial fluid of OA patients (Zhang et al., 2018). Combining human OA synovium with OA cartilage explant culture increased cartilage expression of catabolic markers such as MMP-13, and cartilage matrix degradation. Moreover, the addition of human OA synovium to cartilage reduced viability in chondrocytes and progressively reduced GAG content (Topoluk et al., 2018), or decreased GAG production in cartilage. However, in the latter study GAG release was not affected (Beekhuizen et al., 2011). Although this does not match the classical concept of inflammation-induced matrix degradation, it is in line with several *in vivo* studies showing that inhibition of synovial inflammation in OA does not improve cartilage integrity (Tellegen et al., 2018; Rudnik-Jansen et al., 2019). However, using the same type of co-culture in another study the presence of OA synovium did induce an increase of GAG release (Hardy et al., 2002). The catabolic/anti-anabolic cues of OA synovial tissue may be related to the presence of macrophages, as their depletion from human OA synovial tissue not only reduced levels of IL-1β in co-culture, but also reduced catabolic responses such as MMP-13 increase (Topoluk et al., 2018).

Using human OA tissue, also a differential effect of the corticosteroid triamcinolone acetonide (TAA) was shown in co-culture. TAA prevented the decrease in GAG production induced by the presence of synovium, while in cartilage monoculture it decreased GAG production (Beekhuizen et al., 2011). Using such an OA tissue based co-culture system, IL-1β-induced cartilage proteoglycan degradation was shown to correlate with induction of COX-2 expression and PGE_2_ production in the synovium. A selective COX-2 inhibitor, SC-236, blocked this degradation in the co-culture, and its effect was reversed by exogenous PGE_2_. (Hardy et al., 2002). The relevance of this finding to OA, however, was not entirely clear, as COX-2 protein could not be detected in unstimulated co-culture. Generally, it should be questioned whether additional inflammatory stimulation with IL-1β would be meaningful, as such co-cultures are fully based on OA tissue. Also meniscal tissue was affected by addition of synovium from patients with early stage OA, resulting in higher expression of inflammatory markers such as IL-6 and IL-8, catabolic markers such as MMP-3 and MMP-10 and GAG release (Favero et al., 2019). Thus, co-culture of cartilage or meniscus with synovium demonstrated that co-culturing influences the degenerative and inflammatory state of the cartilaginous tissues involved, mimicking OA *in vivo*. However, using bovine tissue, also healthy synovium induced GAG release and collagen II degradation in healthy cartilage, which suggests that all of these models are equally valid (Siebuhr et al., 2020).

Addition of healthy equine synovium to healthy equine osteochondral explant (Figure 4D) culture increased collagen II expression in the cartilage (Haltmayer et al., 2019). While IL-1β stimulation increased secretion of TNF-α and MMP-13 by cartilage in both monoculture and co-culture, the increase was attenuated in the co-culture, suggesting a protective effect of the synovium tissue (Byron and Trahan, 2017). In contrast, a set of stimuli comprising mechanical injury, IL-1β and TNF-α addition induced a significantly higher increase in MMP-1 gene expression in co-culture with healthy equine synovium compared to equine osteochondral explants alone (Haltmayer et al., 2019). Moreover, these stimuli induced a shift towards the more inflammatory M1 type in synovial macrophages, which mimicked the *in vivo* situation closer than the corresponding model of osteochondral explants alone (Haltmayer et al., 2019). The lack of protection by the healthy synovium in the latter study may be explained by the impact of the combined stimuli that may have been too large to overcome.

Also the joint capsule with the fibrous outer layer has been used in co-culture (Lee et al., 2009; Swärd et al., 2017). Adding healthy bovine synovial capsule tissue to healthy bovine cartilage culture increased MMP-13 and ADAMTS-4 expression in cartilage. Mechanical injury of the cartilage additionally increased ADAMTS-5 and MMP-3 expression as well as ADAMTS- and MMP- mediated digestion of aggrecan in the cartilage (Lee et al., 2009; Swärd et al., 2017) only in the presence of the synovial capsule tissue.

Unfortunately, using osteochondral tissue makes it difficult to pinpoint the role of bone and cartilage. Investigating the interplay and assigning changes with processes in one of the tissues may help revealing the pathological mechanisms involved in OA. However, taking into account that the joint is comprised of multiple tissues, it could also be argued that the more tissue types are included the closer is the model to the *in vivo* situation.

### 4.4 Co-Culture of Other Joint Structures

Apart from bone, cartilage and synovium, other joint structures such as infrapatellar fat pad or innervating tissue are known to influence joint homeostasis, OA onset and progression (Clements et al., 2009; Cai et al., 2015; Kim et al., 2016). While no effect was observed of adding bovine healthy fat pad tissue to bovine meniscus explant culture, it did increase GAG release in cartilage explant culture (Nishimuta et al., 2017). As net GAG content within the cartilage was not altered, GAG release may have been attributed to increased GAG production, suggesting that the infrapatellar fat may also have beneficial effects (Nishimuta et al., 2017). However, addition of OA IPFP-derived conditioned medium induced proteoglycan and collagen II loss in preserved cartilage from OA patients and an increase of MMP-3 and COX-2 positive cells (Zhou et al., 2020). Additionally, conditioned medium from OA-derived IPFP increased p38MAPK and ERK1/2 signaling in human chondrocytes significantly inducing the upregulation of MMPs, ADAMTS-4 as well as IL-1β, IL-6 and COX-2 in chondrocytes. (Zhou et al., 2020). These results indicate the role of IPFP as a causative factor in OA. This is in line with an *in vivo* study in which lipodystrophic mice on a high-fat diet were protected from OA development and lost this protection upon fat transplantation (Collins et al., 2021). Possibly early pathological changes convert the positive effect of the IPFP in a healthy joint to a negative influence.

Clinically, pain is a major burden in OA, but the mechanisms involved in the generation of pain are still poorly understood. To investigate the influence of inflamed synovium on nervous tissue, synovial tissue from healthy human donors or osteoarthritic patients was co-cultured with dorsal root ganglia (DRG) obtained from healthy rats (Li et al., 2011). In co-culture with human osteoarthritic synovium, rat DRGs showed increased expression of the neurokinin substance P and its receptors NK1 and NK2, indicative of increased nerve stimulation (Li et al., 2011). Moreover, expression of neuropeptide Y receptor, which has been shown to be associated with chronic pain (Upadhya et al., 2009), was increased upon co-culture with OA synovium, but not healthy synovium or DRG culture alone, delineating the role of the synovium in OA pain as already indicated *in vivo* (Im et al., 2010; Li et al., 2011). However, as tissues were from rat and human origin, whether this is observed in human/human co-culture remains to be elucidated.

### 4.5 Drug Development in Tissue-Co-Culture Systems

Co-culture systems have also been used for drug development. So far, various types of treatments, ranging from drugs to platelet-rich plasma and stem cells to biomechanics-changing additives were investigated in OA models (Table 4). Most of the co cultures systems used to this end have been based on the use of synovial and cartilage tissues or cells.

**TABLE 4:** Summary of effects of drugs in mono and co-cultures.

Model	Species	Cell/tissue	Additional stimulus	Treatment	Treatment effect on monoculture	Treatment effect on co-culture	References
chondrocytes and synoviocytes	human	chondrocytes	IL-1β	NAPA		NFκB pathway activity↓	Pagani et al. (2019)
chondrocytes in gelatin and LPS-activated macrophages	porcine	chondrocytes		celecoxib		MMP1,-3, PGE_2_ ↓	Peck et al. (2014)
chondrocytes in alginate and sclerotic osteoblasts	human	chondrocytes		carnosol pre-treated osteoblasts		aggrecan production ↑, MMP-3, ADAMTS-4, -5 ↓	Sanchez et al. (2015)
		osteoblasts		carnosol	IL-6 and PGE_2_↓		
joint-on-a-chip (fibrin-based)	human	HUVEC	chemokine mix (CCL 2-5)	chemokine receptor antagonist		monocyte extravasation↓	Mondadori et al. (2021)
cartilage and synovium	bovine	cartilage	IL-1α	IL1 receptor antagonist	GAG & collagen loss↘	GAG & collagen loss ↓↓	Mehta et al. (2019)
	bovine	cartilage		ADAMTS-5 targeting nanobody		GAG loss ↓	Siebuhr et al. (2020)
	canine	cartilage	IL-1	hyaluronic acid		MMP-3 ↓, GAG content ↑	Greenberg et al. (2006)
OA cartilage and OA synovium	human	cartilage		MSCs	none	GAG ↑, chondrocyte viability ↑	Topoluk et al. (2018)
		cartilage		triamcinolone acetonide	GAG production ↓	GAG production ↗	Beekhuizen et al. (2011)
		cartilage		amniotic fluid or PRP		ADAMTS-5, TIMP-1 ↓, aggrecan ↑	O'Brien et al. (2019); Osterman et al. (2015)
		synovium	IL-1β			ADAMTS-5, TIMP-1 ↓	
cartilage and joint capsule	ovine	cartilage	LPS	S-(+)-ibuprofen	NO, aggrecan loss ↓↓	NO, aggrecan loss ↘	Bédouet et al. (2015)

An insert-based co-culture system of IL-1β stimulated human chondrocytes and synoviocytes was used to look at the effect of the N-acetyl phenylalanine glucosamine derivative (NAPA) as a drug targeting the NFκB pathway involved in OA. The addition of synoviocytes to chondrocytes induced increased phosphorylation on serine 10 in histone 3, suggesting higher NFκB pathway activity and a shift to a more inflammatory state (Pagani et al., 2019). NAPA treatment was able to reduce the phosphorylation in the co-culture. Whether the co-culture model better mimics the *in vivo* drug response was not explained as no comparison of NAPA treatment on mono and co-culture was performed (Pagani et al., 2019). Even though chondrocytes and synoviocytes from both healthy and OA tissue were available, both cell sources were used interchangeably. However, most likely the strong stimulation with IL-1β would not have allowed for the detection of any differences. In combination with LPS stimulation, addition of sheep synovial capsule tissue to cartilage weakened the protective effect of S-(+)-ibuprofen on nitric oxide synthesis and aggrecan loss, possibly related to the higher levels of PGE_2_ produced by the synovium (Bédouet et al., 2015). In contrast, the combination with synovial tissue eliminated the requirement for continuous presence of IL-1 receptor antagonist in order to prevent GAG and collagen loss and improve chondrocyte viability in IL-1α-stimulated cartilage explants (Mehta et al., 2019). The discrepancy in the role of the synovial tissue may lie in the type of stimulus, with LPS possibly inducing a more general and strong inflammatory response, also in the synovium via Toll Like receptors, compared to IL-1α. Principal component analysis of secretome data from the co-culture in comparison with respective monocultures revealed distinct clustering between the culture setups, which indicated crosstalk between cartilage and synovium (Mehta et al., 2019).

IL-1β stimulated co-cultures of cartilage and synovium from OA patients were also used to study the therapeutic effect of PRP and amniotic viscous fluid. Both significantly reduced ADAMTS-5 and TIMP-1 expression in both cartilage and synovium, back to levels seen in non-stimulated cartilage, and increased aggrecan expression in cartilage, although only PRP also reduced nitric oxide production (Osterman et al., 2015; O'Brien et al., 2019). Unfortunately, the added value of co-culture was not addressed in either study. Several recent clinical trials furthermore failed to show any effect on joint integrity and clinical outcomes of PRP treatment compared to placebo (Hohmann et al., 2020; Bennell et al., 2021). Also hyaluronic acid (HA), a key component of the synovial fluid, has been used for treating inflammation and pain in OA as so called visco-supplementation. Commercially available hyaluronic acid (Hyalgan or Synvisc) added to IL-1-stimulated canine synovium-cartilage co-cultures inhibited the loss of GAG content in cartilage, and reduced MMP-3 expression if Hyalgan was added (Greenberg et al., 2006). Yet, the *in vitro* effects of these biologicals are in contrast with the poor level of evidence of efficacy of HA treatment (Lin et al., 2019). This may further point towards the limited value of IL-1 stimulation *in vitro* models of OA, at the concentrations commonly used, which are between 100–1000 fold higher than those found in OA patients (Calich et al., 2010; Vangsness et al., 2011; Tsuchida et al., 2014). The IL-1 levels in the pg/ml range found in the synovial fluid of these patients, together with the increased concentrations of its natural inhibitor IL-RA may also explain why several clinical trials failing to show an effect of IL-1 inhibition in the treatment of OA (Kahle et al., 1992; Irie et al., 2003; Tsuchida et al., 2014). Another emerging OA treatment is the use of mesenchymal stem cells. Amniotic and adipose tissue derived MSCs were able to increase GAG content and chondrocyte viability and prevent an increase in OARSI score in cartilage, only in synovium cartilage co-culture from OA patients. (Topoluk et al., 2018). It was, however, not clear what processes in the synovial tissue were responsible for this modulating effect.

More recent approaches also investigated the use of an ADAMTS-5 targeting nanobody (Nanobody® M6495), which was able to dose-dependently inhibit the bovine synovium-induced GAG breakdown in bovine cartilage explants and in human OA cartilage monoculture (Siebuhr et al., 2020). This indicated potential as a treatment for OA, although for human OA cartilage proof of effectivity was obtained only in cartilage stimulated with high doses of proinflammatory cytokines.

Non-sclerotic and sclerotic osteoblasts were used in co-culture with chondrocytes to study the effect of carnosol, a polyphenol extracted from rosemary. Pretreatment of sclerotic osteoblasts with carnosol could prevent and even partially reverse the reduced aggrecan production by chondrocytes induced by untreated sclerotic osteoblasts. Carnosol pretreatment furthermore decreased MMP-3, ADAMTS-4 and ADAMTS-5 in chondrocytes compared to co-culture with non-pretreated osteoblasts. However, the reduced collagen II gene expression in chondrocytes could not be mitigated by pretreatment (Sanchez et al., 2015).

## 5 Mechanisms for Interactions Between Joint Tissues

Several mechanisms have been postulated to be operational in the crosstalk between joint tissues. Cells within synovium and cartilage secrete cytokines which are also found in the synovial fluid resulting in inflammation and cartilage degradation (Goldring and Otero, 2011). Indirect evidence for the role of cytokines in this crosstalk is the similarity of cytokine levels in co-cultures of human OA cartilage and synovial tissue to OA synovial fluid (Rutgers et al., 2010) and the observation that an antibody neutralizing oncostatin M in synovial fluid could counteract the inhibition of matrix production in synovial fluid-exposed cartilage tissue (Beekhuizen et al., 2013a). Also the prostaglandins that are derived from unsaturated fatty acids were shown to act as proinflammatory mediators. PGE_2_ produced by the human OA synovium correlated with proteoglycan degradation in OA cartilage (Hardy et al., 2002). Extracellular vesicles (EVs) may also play a role in OA progression, by carrying proteins, mRNA and even DNA from one tissue to another (Figure 5) (Miyaki and Lotz, 2018). *In vitro*, exosomes produced by IL-1β stimulated human synovial fibroblasts induced increased expression of MMP-13 and ADAMTS-5 and decreased ACAN and COL2A1 gene expression in human chondrocytes, as well as increased proteoglycan release in rat cartilage. Analysis of the exosomes showed differential expression of 50 miRNAs, which may have contributed to the EV induced changes (Kato et al., 2014). Gap junctions are also found in synovium and other joint tissues and there expression is altered in OA joint (Donahue et al., 2018). *In vitro* data also showed gap junction mediated calcium signaling between synovial cells and chondrocytes in co-culture (D'Andrea et al., 1998) or gap junction formation between chondrocytes and osteoblasts. Their contribution to OA, however, remains to be explained. Cell-cell-contacts *via* gap junctions between chondrocytes and osteocytes have not been confirmed *in vivo* so far. Even though it is well established that osteocytes communicate via gap junctions consisting of connexins (Doty, 1981), connexins expressed in chondrocytes are thought to rather function as hemichannels which are not coupled to another cell but fulfil other roles (Plotkin and Stains, 2015).

## 6 Considerations and Discussion

Co-cultures of joint cells and tissues have been widely used in OA research and have replaced animal models to a certain extent. Essential to their applicability is the degree to which they reflect the *in vivo* processes in OA, in terms of cartilage metabolism, inflammation and the response of these parameters to pathophysiological stimuli or drugs. However, this is not always easy to define, as our capacity to real-time detect processes occurring in OA is still limited. Crosstalk between co-cultured cells or tissues, evidenced by differential behavior compared to their respective monocultures, may further indicate the relevance of a co-culture system. However, as shown for the degenerative effect of healthy bovine synovial tissue on healthy cartilage tissue, this still may not always reflect the *in vivo* conditions. Several explanations may be given for this phenomenon. First of all, the presence of other joint tissues may be required for maintenance of homeostasis. Also the process of cutting tissue and inserting it into a novel biochemical and mechanical environment may affect behavior, as was shown by the peak in cytokine production by cartilage explants immediately after their isolation (Beekhuizen et al., 2011). Also the ratio of one versus the other tissue may affect the response as was shown in cell and cell/tissue co-culture (Larsson et al., 2011). Achieving the optimal ratio mimicking the *in vivo* joint will pose a challenge that nevertheless may be worthwhile taking. Finally of course it is possible that the assumed *in vivo* interactions do not occur, or to a different extent. The complexity of co-culture models comes with several tradeoffs. Monolayer co-cultures may yield insightful and detailed information on the interplay of cell types. However, cultured cells often lose their *in vivo* phenotype*.* Bioengineered models, utilizing hydrogel-encapsulated cells may at least offer a more native microenvironment, especially for bone and cartilage cells and can help investigating to what extent neighboring cells and tissues influence each other in a 3D environment. Biomechanical properties of the hydrogel should always be evaluated as stiffness or porosity of the hydrogel might differ from *in vivo* (Bao et al., 2020). Tissue explants provide the native tissue structure to the cells. Patient-derived tissue explants are a good resource for co-culture systems as they reflect these characteristics and are derived from spontaneously degenerated tissue. However, in addition to the limited availability of such tissues, OA is a disease with different phenotypes, where mechanical stress, inflammation and degeneration play roles of varying importance. Hence, tissue properties vary highly between donors. Therefore animal-derived explants have also often been utilized. Here, culture conditioned are better controlled yielding in higher reproducibility. Yet, a clear drawback of animal tissue is that joint physiology might differ from human and that physical characteristics such as joint size and cartilage thickness cannot be matched optimally to human OA joint tissue (Singh et al., 2021). Another distinct disadvantage of this approach is that these tissues originate from young and healthy animals, and therefore OA has to be induced *in vitro*. Stimuli such as cytokines or mechanical stress are frequently used to this end. However, care must be taken that the stimulus is physiological, which currently often is not the case for several proinflammatory cytokines used to induce OA, commonly at supraphysiological concentrations (Beekhuizen et al., 2013b). Using cytokine concentrations of the synovium of OA joints, possibly even in more complex mixes, can help to make the stimulus more pathophysiological (Beekhuizen et al., 2013a) and thereby prevent ineffective treatments being developed such as those based on IL-1 inhibition. Still, any response observed will still be generated using young tissue and cells if animal tissues are used and therefore human tissues remain the source of choice. Possibly the type of stimulus may be adapted to the research question. If anti-inflammatory drugs are tested, the stimulus may be based on inflammatory characteristics. This would imply that depending on the research question, the model does not need to mimic all features of OA. However, several crossroads in the pathological mechanisms of OA have been described, so care must be taken to thoroughly characterize existing and novel co-culture models based on OA induction by external stimuli. More systematic comparisons of co-cultures with their respective monocultures and their correlation to *in vivo* data will be indispensable here. Finally, most models described in the current review consisted of only two different compartments, while the joint comprises many tissues. Platforms such as joint-on-a-chip systems may enable the combination of multiple joint compartments while allowing to evaluate their individual roles, provided the cell phenotype stays similar to in the joint *in vivo*. Here, any issues concerning human cell availability may be counteracted by making use of induced pluripotent stem cell (iPS) technology, which has advanced recently to the possibility of generating different cell types.

## 7 Conclusion

The utilization of co-culture models is key to reduce the animal use in OA research and to gain more understanding of the interplay in OA joints at the cell and tissue level. This can be a useful tool in drug development, but also in other research questions such as the role of biomechanical loading. The model should reflect respective characteristics of and processes in OA. Better validation of models is key here. Moreover, ideally more complex models incorporating more tissues and cell types are introduced.
